# Impact of Glucagon‐Like Peptide‐1 Receptor Agonists on the Dementia Incidence in Patients With Type 2 Diabetes Mellitus: A Population‐Based Longitudinal Cohort Study

**DOI:** 10.1002/dmrr.70058

**Published:** 2025-06-07

**Authors:** Hung‐Wen Cheng, Shun‐Fa Yang, Pei‐Lun Liao, Chiu‐Hsian Lee, Gwo‐Ping Jong

**Affiliations:** ^1^ Institute of Medicine Chung Shan Medical University Taichung Taiwan; ^2^ Department of Pharmacy Chung Shan Medical University Hospital Taichung Taiwan; ^3^ Department of Medical Research Chung Shan Medical University Hospital Taichung Taiwan; ^4^ Department of Nursing Chung Shan Medical University Taichung Taiwan; ^5^ Division of Cardiology Department of Internal Medicine Chung Shan Medical University Hospital Taichung Taiwan

**Keywords:** dementia, glucagon‐like peptide‐1 receptor agonists (GLP‐1RAs), type 2 diabetes mellitus (T2DM)

## Abstract

**Aims:**

Type 2 diabetes mellitus (T2DM) is recognized for increasing the risk of dementia; however, conclusive evidence supporting interventions to mitigate this risk remains elusive. This study endeavours to ascertain whether the glucagon‐like peptide‐1 receptor agonists (GLP‐1RAs) correlate with reduced incidence of dementia.

**Materials and Methods:**

The cohort comprised individuals initiating treatment with either GLP‐1RAs or non‐GLP‐1RAs medications between 2013 and 2021. This study examined the association between GLP‐1RAs and the risk of all‐cause dementia. Propensity score‐matched and Cox proportional hazard models were employed to calculate the adjusted hazard ratio (aHR) and confidence interval (CI) for the incidence of dementia.

**Results:**

Among a cohort comprising 109,778 individuals, the use of GLP‐1RA demonstrated a reduced risk of dementia compared with its non‐use (aHR, 0.90; 95% CI, 0.83–0.97). Subgroup analyses stratified by different diabetic complications revealed significantly lower dementia incidence rates among GLP‐1RAs users than among non‐GLP‐1RAs users. Individuals aged ≤ 75 years demonstrated a significant protective effect within GLP‐1RAs users.

**Conclusions:**

The utilization of GLP‐1 receptor agonists instead of non‐GLP‐1RAs medications demonstrated an association with a decreased incidence of dementia.

## 1 Introduction

Dementia represents a rapidly growing global health challenge, with > 50 million individuals affected worldwide as of 2016. This number may triple by 2050, owing to increasing life expectancies [1, 2, 3]. It stands as a leading cause of disability among older people. Despite new monoclonal antibody treatments aimed at slowing dementia progression, their use may be limited by severe adverse reactions. Numerous studies have indicated a link between type 2 diabetes mellitus (T2DM) and increased risk of cognitive decline and dementia [4, 5, 6]. The co‐occurrence of T2DM and dementia imposes a substantial economic burden on both affected families and healthcare systems. Consequently, mitigating the effect of dementia incidence in individuals with T2DM emerges as a critical health priority.

Previous studies have highlighted the importance of achieving optimal glycaemic control and minimising diabetic complications in reducing dementia risk [7, 8]. Moreover, studies have demonstrated that glucagon‐like peptide‐1 receptor agonists (GLP‐1RAs) contribute to favourable glycaemic control, weight loss, and renal protection [9, 10, 11, 12]. Despite these findings, the precise association between GLP‐1RAs and dementia incidence remains vague. Thus, we undertook a retrospective cohort study to elucidate the relationship between GLP‐1RAs and dementia incidence within the broader Taiwanese population. This study aimed to discern whether the utilization of GLP‐1RAs correlates with incident dementia risk in a cohort of Taiwanese patients diagnosed with T2DM.

## 2 Materials and Methods

### 2.1 Study Design

This retrospective case–control cohort study utilised insurance claims data sourced from the Taiwanese Bureau of National Health Insurance (TBNHI) spanning from January 2013 to December 2021. This study was approved by the ethics committee of Chung Shan Medical University Hospital (CS2‐24009). Written consent was not sought from the study participants as only de‐identified data were obtained from the TBNHI. The ethics committee waived the need for patient consent for this study.

### 2.2 Study Population

GLP‐1RAs users were identified as patients who first received prescriptions for these GLP‐1RAs continuously for > 3 months during the study period, with the respective index date being the initial use of GLP‐1RAs day by an individual. Conversely, non‐users of GLP‐1RAs were characterised as patients with index date who did not receive prescriptions for these GLP‐1RAs throughout the study period.

The identification of diagnosed cases of T2DM relied on diagnostic codes derived from the International Classification of Diseases, ninth and 10th Revisions, Clinical Modification (ICD‐9‐CM and ICD‐10‐CM, respectively). Newly diagnosed T2DM was defined as the first instance of a T2DM code appearing in outpatient or inpatient claim records between 2013 and 2021. The specific ICD‐9 and ICD‐10 codes utilised for defining the inclusion criteria for patients with T2DM, study events, and comorbidities are detailed in Supporting Information S1: Table S1.

**FIGURE 1:**
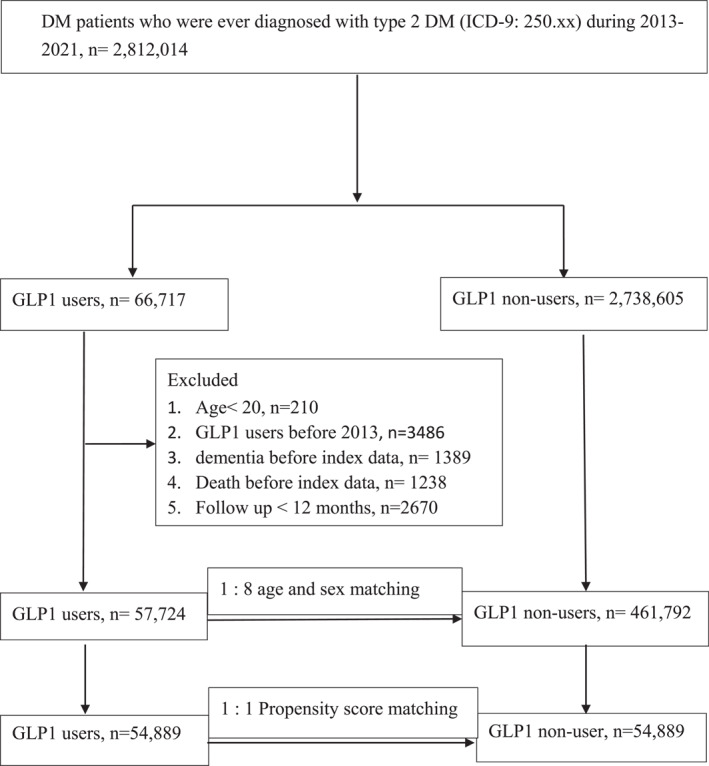
Patient flowchart.

Patients' exclusion criteria were as follows: (1) aged < 20 years, (2) use of GLP‐1 RAs before 2013, and (3) a diagnosis of dementia before the index date. Given the disparities in baseline characteristics and dementia risk between GLP‐1RAs users and non‐users, propensity score matching (PSM) was applied. Patients were matched by age, sex, comorbidities and drug index date, resulting in a final ratio of 1:1 for patients with T2DM who were users and non‐users of GLP‐1RAs (Figure 1).

### 2.3 Drug Exposure

The study considered medications including GLP‐1RAs (such as exenatide, liraglutide, dulaglutide, and semaglutide) and other antidiabetic medications (e.g., linagliptin, saxagliptin and sitagliptin), all covered under TBNHI. Given the chronic and irreversible nature of dementia, the primary analysis employed an ‘intention‐to‐treat’ approach, wherein data were not censored beyond the initiation of alternative medications (i.e., crossover) or discontinuation of baseline treatments.

### 2.4 Outcome

The outcome measure was the incidence of dementia, which was defined by the presence of relevant ICD‐9‐CM and ICD‐10‐CM codes recorded in either outpatient or inpatient department records at least once between 2013 and December 31, 2021. The index date for the use of GLP‐1RAs was based on the first day of prescription, matching the same day as that for non‐GLP‐1RAs users, and the observation window commenced at least 1 year from cohort entry. GLP‐1RAs have been covered by TBNHI prescriptions since 2013 and were utilised until the end of the study (31 December 2021). Patients were followed up from their first prescription of GLP‐1RAs until the occurrence of dementia or the end of the study. Analysis was conducted according to patients' original group assignment, irrespective of adherence or duration of GLP‐1RAs use. This approach ensured consistency and minimised potential biases in the study outcome analysis.

### 2.5 Study Variables

The following variables were identified as potential confounders: index month, sex, age, comorbidities, and concurrent medications. Age was treated as both a continuous variable and categorised into groups: 20–49, 50–59, 60–69, or ≥ 70 years. Concurrent medications encompassed nonsteroidal inflammatory drugs, corticosteroids, aspirin, statins, other antidiabetic agents, alpha‐ or beta‐blockers, calcium channel blockers, angiotensin‐converting enzyme inhibitors, and angiotensin receptor blockers (Supporting Information S1: Table S2). Comorbidities were defined using ICD‐9‐CM and ICD‐10‐CM codes (Supporting Information S1: Table S1). These comorbidities comprised hypertension, hyperlipidaemia, heart failure, chronic kidney disease, liver disease, chronic pulmonary diseases, malignancy, urinary tract infection, asthma, coronary artery disease, obstructive sleep apnoea, atrial fibrillation/flutter, alcohol‐related disorders, depression, rheumatoid arthritis, and stroke.

### 2.6 Statistical Analysis

All analyses were performed using SAS 9.4 Statistical Software (SAS Institute Inc., Cary, NC, USA). Data were presented as valid percentages and mean values with standard deviations. To address confounders, PSM was employed based on a logistic regression model. The absolute standardized difference (ASD) was utilised to assess the balance of baseline characteristics between the study groups, and covariates achieving an absolute standardized difference of < 0.1 were considered adequately balanced [13].

Initially, the patients were matched 1:8 based on age and sex. Another PSM model was utilised to compare the effects of the two study groups on the study outcome. Adjusted hazard ratios (aHRs) and 95% confidence intervals (CIs) were calculated, accounting for key risk factors associated with dementia development, including age, sex, comorbidities, and concurrent medications. The risk of study outcomes over time for GLP‐1RAs users compared with non‐GLP‐1RAs users was assessed using survival analysis via the Kaplan–Meier method. This method was employed to visualise the cumulative incidence proportions of each exposure group over time and evaluate any significant time‐dependent trends in relative hazards. Multiple Cox regression was employed to compare the risk of developing incident dementia between GLP‐1RAs users and non‐GLP‐1RAs users. All effects were analysed utilising an intention‐to‐treat approach.

Sensitivity analyses were performed to evaluate the robustness of the study's findings. Patients with newly diagnosed T2DM were considered to assess the effect on incident dementia outcomes in the sensitivity analyses. Subgroup analyses stratified by sex, age, duration and severity of T2DM were performed on the outcomes. Statistical significance was defined at *p*‐value <  0.05.

## 3 Result

### 3.1 Population Characteristics

Out of 109,778 patients diagnosed with T2DM, 54,889 commenced treatment with GLP‐1RAs, and an equal number initiated treatment with non‐GLP‐1RAs (Figure 1) between January 2013 and December 2020. Detailed characteristics for each treatment group are presented in Table 1, with all covariates demonstrating a standardized mean difference of < 0.1 after PSM. Further information regarding each drug class is provided in Supporting Information S1: Table S2.

**TABLE 1:** Baseline characteristics of all patients.

	8:1 sex age matching			After PSM		
	Non‐ GLP‐1	GLP‐1	ASD	Non‐ GLP‐1	GLP‐1	ASD
*N*	439,680	54,960		54,889	54,889	
Index year			0.0000			0.0348
2013	11,920 (2.71%)	1490 (2.71%)		1702 (3.10%)	1489 (2.71%)	
2014	18,456 (4.20%)	2307 (4.20%)		2420 (4.41%)	2305 (4.20%)	
2015	25,864 (5.88%)	3233 (5.88%)		3302 (6.02%)	3230 (5.88%)	
2016	31,088 (7.07%)	3886 (7.07%)		3886 (7.08%)	3882 (7.07%)	
2017	72,736 (16.54%)	9092 (16.54%)		8944 (16.29%)	9082 (16.55%)	
2018	101,944 (23.19%)	12,743 (23.19%)		12,670 (23.08%)	12,725 (23.18%)	
2019	107,240 (24.39%)	13,405 (24.39%)		13,247 (24.13%)	13,381 (24.38%)	
2020	70,432 (16.02%)	8804 (16.02%)		8718 (15.88%)	8795 (16.02%)	
Sex			0.0000			0.0012
Female	214,368 (48.76%)	26,796 (48.76%)		26,729 (48.70%)	26,761 (48.75%)	
Male	225,312 (51.24%)	28,164 (51.24%)		28,160 (51.30%)	28,128 (51.25%)	
Age			0.0000			0.0263
20–49	131,731 (29.96%)	16,748 (30.47%)		17,285 (31.49%)	16,711 (30.45%)	
50–59	119,106 (27.09%)	14,740 (26.82%)		14,774 (26.92%)	14,722 (26.82%)	
60–69	122,165 (27.78%)	15,167 (27.60%)		14,851 (27.06%)	15,159 (27.62%)	
≥ 70	66,678 (15.17%)	8305 (15.11%)		7979 (14.54%)	8297 (15.12%)	
Mean (± SD)	56.32 (13.04)	56.22 (13.08)		56.18 (12.64)	56.22 (13.08)	
Urbanization			0.1027			0.0000
Urban	254,192 (57.81%)	34,451 (62.68%)		34,514 (62.88%)	34,396 (62.66%)	
Sub‐urban	142,413 (32.39%)	15,566 (28.32%)		15,487 (28.22%)	15,554 (28.34%)	
Rural	43,075 (9.80%)	4943 (8.99%)		4888 (8.91%)	4939 (9.00%)	
Insurance property			0.0605			0.0000
Public insurance	14,517 (3.30%)	1921 (3.50%)		1885 (3.43%)	1917 (3.49%)	
Labour insurance	258,034 (58.69%)	32,912 (59.88%)		33,164 (60.42%)	32,864 (59.87%)	
F.W.F insurance	62,978 (14.32%)	6764 (12.31%)		6666 (12.14%)	6760 (12.32%)	
Other	104,151 (23.69%)	13,363 (24.31%)		13,174 (24.00%)	13,348 (24.32%)	
DCSI			0.4340			0.0000
0	232,184 (52.81%)	18,339 (33.37%)		18,262 (33.27%)	18,331 (33.40%)	
1–2	158,817 (36.12%)	25,108 (45.68%)		25,401 (46.28%)	25,067 (45.67%)	
≥ 3	48,679 (11.07%)	11,513 (20.95%)		11,226 (20.45%)	11,491 (20.93%)	
Comorbidities						
Hypertension	237,077 (53.92%)	34,062 (61.98%)	0.1637	33,832 (61.64%)	34,015 (61.97%)	0.0069
Hyperlipidaemia	249,063 (56.65%)	38,066 (69.26%)	0.2635	38,381 (69.92%)	38,005 (69.24%)	0.0149
Heart failure	13,150 (2.99%)	2920 (5.31%)	0.1166	2864 (5.22%)	2915 (5.31%)	0.0042
CKD	51,627 (11.74%)	10,592 (19.27%)	0.2092	10,353 (18.86%)	10,564 (19.25%)	0.0098
Liver disease	57,069 (12.98%)	7341 (13.36%)	0.0112	7418 (13.51%)	7336 (13.37%)	0.0044
COPD	18,993 (4.32%)	2734 (4.97%)	0.0311	2725 (4.96%)	2729 (4.97%)	0.0003
Malignancy	25,669 (5.84%)	2848 (5.18%)	0.0288	2840 (5.17%)	2848 (5.19%)	0.0007
UTI	47,852 (10.88%)	7205 (13.11%)	0.0686	7141 (13.01%)	7194 (13.11%)	0.0029
Asthma	19,335 (4.40%)	3321 (6.04%)	0.0740	3220 (5.87%)	3311 (6.03%)	0.0070
CAD	54,357 (12.36%)	9749 (17.74%)	0.1508	9610 (17.51%)	9731 (17.73%)	0.0058
Obstructive sleep apnoea	2624 (0.60%)	759 (1.38%)	0.0793	668 (1.22%)	752 (1.37%)	0.0135
Atrial fibrillation and flutter	5494 (1.25%)	908 (1.65%)	0.0337	941 (1.71%)	908 (1.65%)	0.0047
Alcohol‐related disorders	5834 (1.33%)	561 (1.02%)	0.0284	574 (1.05%)	561 (1.02%)	0.0023
Depression	48,119 (10.94%)	5900 (10.74%)	0.0067	5788 (10.54%)	5893 (10.74%)	0.0062
RA	3457 (0.79%)	495 (0.90%)	0.0125	463 (0.84%)	494 (0.90%)	0.0061
Stroke	23,103 (5.25%)	3075 (5.59%)	0.0150	4053 (7.38%)	3070 (5.59%)	0.0728
Medication						
NSAIDs	300,411 (68.32%)	38,483 (70.02%)	0.0367	38,343 (69.86%)	38,434 (70.02%)	0.0036
Corticosteroids	118,336 (26.91%)	16,264 (29.59%)	0.0595	16,159 (29.44%)	16,239 (29.59%)	0.0032
Aspirin	97,000 (22.06%)	16,518 (30.05%)	0.1829	16,317 (29.73%)	16,496 (30.05%)	0.0071
Statin	237,315 (53.97%)	41,410 (75.35%)	0.4587	41,449 (75.51%)	41,340 (75.32%)	0.0046
Biguanides	313,810 (71.37%)	46,774 (85.11%)	0.3375	47,193 (85.98%)	46,704 (85.09%)	0.0253
Sulfonylureas	153,675 (34.95%)	25,756 (46.86%)	0.2441	25,746 (46.91%)	25,703 (46.83%)	0.0016
Alpha glucosidase inhibitors	51,745 (11.77%)	12,968 (23.60%)	0.3138	12,739 (23.21%)	12,924 (23.55%)	0.0080
Thiazolidinediones	54,147 (12.32%)	12,870 (23.42%)	0.2929	12,639 (23.03%)	12,822 (23.36%)	0.0079
DPP‐4 inhibitors	105,843 (24.07%)	24,289 (44.19%)	0.4343	24,718 (45.03%)	24,232 (44.15%)	0.0178
Insulin	82,184 (18.69%)	31,841 (57.93%)	0.8823	31,365 (57.14%)	31,770 (57.88%)	0.0149
SGLT‐2 inhibitors	39,029 (8.88%)	12,934 (23.53%)	0.4059	12,553 (22.87%)	12,874 (23.45%)	0.0139
Alpha‐blockers	18,352 (4.17%)	3270 (5.95%)	0.0811	3234 (5.89%)	3266 (5.95%)	0.0025
Beta‐ blockers	123,278 (28.04%)	19,926 (36.26%)	0.1766	19,582 (35.68%)	19,888 (36.23%)	0.0116
CCB	123,962 (28.19%)	16,736 (30.45%)	0.0496	16,566 (30.18%)	16,709 (30.44%)	0.0057
ACEI	30,029 (6.83%)	4301 (7.83%)	0.0382	4370 (7.96%)	4298 (7.83%)	0.0049
ARB	194,182 (44.16%)	32,747 (59.58%)	0.3123	32,494 (59.20%)	32,687 (59.55%)	0.0072

Abbreviations: ACEI, angiotensin‐converting enzyme inhibitors; ARB, angiotensin II receptor blockers; ASD, absolute standardized difference; CAD, coronary artery disease; CCB, calcium channel blockers; CKD, chronic kidney disease; COPD, chronic obstructive pulmonary disease, DPP‐4 inhibitors, dipeptidyl peptidase‐4 inhibitors; F.W.F, farmer, member of water conservancy and fisheries association; GLP‐1, Glucagon‐like peptide‐1 agonists; NSAIDs, nonsteroidal anti‐inflammatory drugs; PSM, propensity score matching; RA, rheumatoid arthritis; SGLT‐2 inhibitors, sodium‐glucose co‐transporter two inhibitors; UTI, urinary tract infection.

### 3.2 | Dementia Risk

During a mean follow‐up period of 41.23 (standard deviation [SD] 21.27) months from cohort entry (i.e., treatment initiation), the crude incidence rate of dementia was 7.20 per 10,000 person‐months (95% CI: 6.87–7.54) for GLP‐1RAs users compared with 6.89 (95% CI: 6.78–7.01) for non‐ GLP‐1RAs users at a 1:8 Ratio. Initially, the use of a GLP‐1RAs was correlated with an increased incidence rate of dementia (HR, 1.05; 95% CI, 1.00–1.10) compared with its non‐user (Table 2). However, subsequent to adjustments for sex, age, comorbidities, and concurrent medication use via multiple Cox regression, GLP‐1RAs users exhibited a reduced risk of incident dementia compared with non‐GLP‐1RAs users (aHR, 0.92; 95% CI, 0.88–0.97) (Table 2).

**TABLE 2:** Incidence rate of Dementia.

	8:1 sex age matching		After 1:1 PSMs	
	Non‐GLP‐1	GLP‐1	Non‐GLP‐1	GLP‐1
*N*	461,792	57,724	54,889	54,889
Follow up person months	19,572,592	2,497,021	1,712,106	1,812,909
New case	13,488	1797	1300	1287
Incidence rate^a^ (95% C.I.)	6.89 (6.78–7.01)	7.20 (6.87–7.54)	7.59 (7.19–8.02)	7.10 (6.72–7.50)
Crude relative risk (95% C.I.)	Reference	1.05 (1.00–1.10)	Reference	0.94 (0.87–1.01)
Adjusted HR^a^ (95% C.I.)^b^	Reference	0.92 (0.88–0.97)	Reference	0.90 (0.83–0.97)

Abbreviation: GLP‐1, glucagon‐like peptide‐1 agonists.

^a^ Incidence rate, per 10,000 person‐month.

^b^ Adjusted hazard ratio, the covariates including year of index, sex, age, co‐morbidities, and medication at baseline.

**FIGURE 2:**
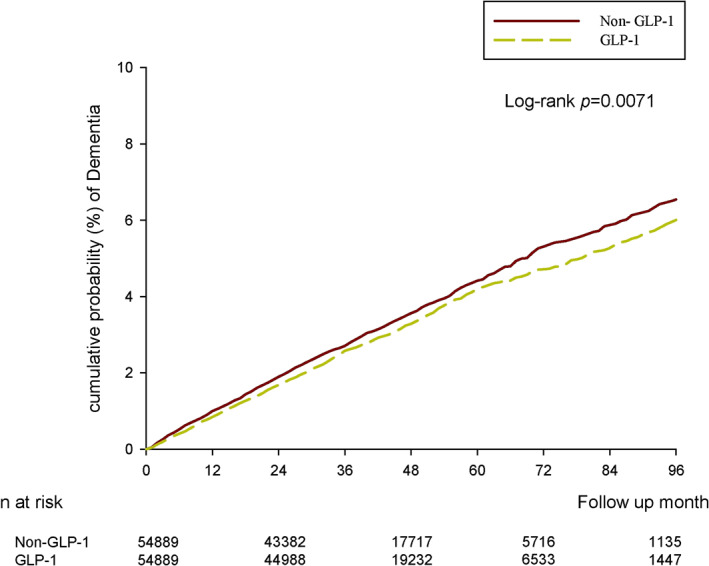
Cumulative risk curve of incidence of dementia for the study cohorts treated with glucagon‐like peptide‐1 receptor agonists versus non‐glucagon‐like peptide‐1 receptor agonist users.

After 1:1 PSM, there was a lower incidence of dementia in the GLP‐1RAs group than in the control group (crude HR: 0.94; 95% CI: 0.87–1.01) (Table 2). Furthermore, the results did not substantially change after adjustments for the index date, sex, age, comorbidities, and concurrent medication at baseline (aHR: 0.90; 95% CI 0.83–0.97). The Kaplan–Meier curves after 1:1 PSM, depicting the cumulative incidence of dementia between GLP‐1RAs users and non‐GLP‐1RAs users, were consistent with the aforementioned results (log‐rank = 0.0071) (Figure 2).

### 3.3 | Sensitivity and Subgroup Analyses

Patients with newly diagnosed T2DM were considered to assess the effect on incident dementia outcomes. After PSM, the covariates (including index years, sex, age, comorbidities, and medications at baseline) of the patients were adjusted to match 89 new cases of dementia among GLP‐1RAs users and 143 new cases of dementia among non‐GLP‐1RAs users for analysis, which also provided similar results (aHR, 0.71; 95% CI, 0.54–0.94; Table 3).

**TABLE 3:** Incidence rate of dementia (newly diagnosed type 2 diabetes mellitus).

	8:1 sex age matching		After PSM	
	Non‐GLP‐1	GLP‐1	Non‐GLP‐1	GLP‐1
Follow up person months	5,054,621	631,754	399,155	630,664
New case	1209	143	143	89
Incidence rate^a^(95% C.I.)	2.39 (2.26–2.53)	2.26 (1.92–2.67)	2.23 (1.81–2.74)	2.27 (1.92–2.67)
Crude relative risk (95% C.I.)	Reference	0.95 (0.80–1.13)	Reference	1.02 (0.78–1.33)
Adjusted HR^a^ (95% C.I.)^b^	Reference	0.79 (0.64–0.96)	Reference	0.71 (0.54–0.94)

^a^ Incidence rate, per 10,000 person‐month.

^b^ Adjusted hazard ratio, the covariates including year of index, sex, age, co‐morbidities, and medication at baseline.

The subgroup analyses revealed similar findings, that is, participants aged < 65 and 65–75 years had significantly decreased incidence of dementia (aHR, 0.87; 95% CI, 0.77–0.98; aHR, 0.90; 95% CI, 0.83–0.99; respectively), but other participants aged > 75 years were aHR, 1.00; 95% CI, 0.90–1.11 (Table 4). In both male and female participants, GLP‐1RAs have demonstrated efficacy. Furthermore, GLP‐1RAs users with severe T2DM displayed lower rates of incident dementia as complications (such as retinopathy, nephropathy, and neuropathy) compared with non‐GLP‐1RAs users over 41.23 months of follow‐up. In patients with T2DM of < 5 years duration and those with a duration of 5–10 years, the use of GLP‐1RAs has been associated with a decreased incidence of dementia (aHR, 0.83; 95% CI, 0.73–0.95; aHR, 0.92; 95% CI, 0.86–0.98, respectively) (Table 4).

**TABLE 4:** Subgroup analysis.

	*N*	Follow up person months	New case	Incidence rate^a^ (95% C.I.)		HR (95% C.I.)	aHR^b^ (95% C.I.)
				Non‐GLP‐1	GLP‐1		
Sex							
Female	241,164	10,210,811	8137	7.90 (7.72–8.09)	8.50 (7.98–9.05)	1.08 (1.01–1.15)	0.93 (0.86–0.99)
Male	253,476	10,181,761	5687	5.58 (5.43–5.73)	5.64 (5.22–6.09)	1.01 (0.93–1.10)	0.89 (0.82–0.98)
Age							
< 65	355,779	15,556,594	3218	2.07 (2.00–2.15)	2.06 (1.86–2.28)	0.99 (0.89–1.10)	0.87 (0.77–0.98)
65–75	106,128	3,845,645	6417	16.56 (16.13–17.00)	17.70 (16.48–19.00)	1.07 (0.99–1.15)	0.90 (0.83–0.99)
> 75	32,733	990,333	4189	41.77 (40.44–43.14)	46.60 (42.71–50.86)	1.12 (1.02–1.23)	1.00 (0.90–1.11)
Duration of type 2 diabetes mellitus (mean = 5.97, SD = 2.52)							
< 5 years	136,523	7,436,430	2978	4.04 (3.89–4.20)	3.68 (3.29–4.12)	0.91 (0.80–1.02)	0.83 (0.73–0.95)
5–10 years	340,711	12,651,702	10,490	8.23 (8.06–8.40)	8.77 (8.30–9.27)	1.07 (1.01–1.13)	0.92 (0.86–0.98)
> 10 years	17,406	304,440	356	11.13 (9.93–12.47)	15.60 (12.11–20.09)	1.40 (1.06–1.85)	1.25 (0.92–1.71)
Comorbidity of type 2 diabetes mellitus							
Retinopathy	137,069	5,764,551	5016	8.72 (8.46–8.99)	8.59 (8.02–9.20)	0.99 (0.92–1.06)	0.91 (0.84–0.98)
Nephropathy	198,465	8,167,873	8029	9.92 (9.69–10.16)	9.36 (8.85–9.90)	0.95 (0.89–1.00)	0.91 (0.85–0.97)
Neuropathy	92,968	3,817,526	5012	13.37 (12.97–13.77)	11.90 (11.08–12.79)	0.89 (0.83–0.97)	0.89 (0.82–0.96)
Other	208,132	8,505,847	3170	3.79 (3.66–3.93)	2.80 (2.38–3.28)	0.74 (0.63–0.87)	0.95 (0.80–1.13)
DCSI							
0	250,523	10,905,100	4155	3.83 (3.72–3.96)	3.51 (3.14–3.94)	0.91 (0.81–1.03)	1.00 (0.88–1.13)
1–2	183,925	7,375,205	5926	8.30 (8.08–8.53)	6.41 (5.95–6.92)	0.77 (0.71–0.84)	0.90 (0.83–0.98)
≥ 3	60,192	2,112,267	3743	18.17 (17.54–18.82)	15.89 (14.73–17.15)	0.88 (0.81–0.95)	0.88 (0.80–0.97)

Abbreviation: DCSI, Diabetes complications severe index.

^a^ Incidence rate, per 10,000 person‐month.

^b^ Adjusted hazard ratio, the covariates including year of index, sex, age, co‐morbidities, and medication at baseline.

## 4 | Discussion

This cohort study demonstrated that new GLP‐1RAs use compared with non‐GLP‐1RAs use was associated with lower dementia risk in people with T2DM. The result was robust in the sensitivity and subgroup analyses. Specifically, among patients with severe T2DM with complications (such as retinopathy, nephropathy, and neuropathy) and T2DM duration < 10 years, GLP‐1RAs users displayed lower rates of incident dementia than non‐GLP‐1RAs users. It appeared that patients with newly diagnosed T2DM may exhibit a more pronounced preventive effect against dementia compared with patients with existing T2DM.

Patients with T2DM have an accelerated rate of cognitive decline and a 1.6‐fold increased risk of dementia development [14, 15]. Important and shared pathological features of T2DM with cognitive decline and dementia, which are characterised by metabolic brain alterations, for example, insulin resistance, altered glucose uptake, and altered glucose utilization. These similarities in pathology are reflected in clinical studies that have demonstrated an increased dementia risk in individuals with T2DM [16, 17, 18].

Among antidiabetic drugs, glucagon‐like peptide‐1 receptor agonists (GLP‐1RAs) play an interesting role in the modulation of neuroinflammation [19, 20, 21]. GLP‐1RAs also play neurotropic and neuroprotective roles in the central nervous system [22]. In addition, GLP‐1RAs reduce Aβ aggregation/deposition and hyperphosphorylation of tau protein, oxidative stress, and neuronal apoptosis, increasing cell proliferation and neurogenesis [23]. In vitro and animal studies have shown that pretreatment with bilateral intrahippocampal injection of liraglutide improves learning and memory deficit in murine ad models [24, 25]. Two clinical studies have revealed a lower risk of all‐cause dementia among GLP‐1RAs users than among non‐GLP‐1RAs users [26, 27].

Using multiple strategies to mitigate bias after adjustments for sex, age, comorbidities, and concurrent medication, the present cohort study strengthened previous findings that GLP‐1RAs use was associated with a lower dementia risk [27, 28, 29]. This study suggests that in patients with T2DM taking GLP‐1RAs, the risk of dementia development is 10% lower than that in non‐users, which may be the possible cause for the increase in its use. Intensive risk factor modification, particularly during midlife (age 45–65 years) was considered to have the potential to delay or prevent a substantial number of dementia cases worldwide [30, 31]. Our cohort study proved that GLP‐1RAs use significantly reduced the incidence of dementia in participants aged < 65 and 65–75 years (aHR, 0.87; 95% CI, 0.77–0.98; aHR, 0.90; 95% CI, 0.83–0.99). GLP‐1RAs reduce the incidence of dementia equally in both sexes, regardless of the duration of T2DM being < 6 or 6–10 years and presence of complications such as retinopathy, neuropathy, and neuropathy. Comparatively, the incidence rate of dementia by GLP‐1RAs type and use versus non‐use of liraglutide and dulaglutide correlated with a decreased incidence of dementia.

In the pooled randomized controlled trial analysis during a median follow‐up of 3.61 years, patients randomized to GLP‐1RAs had a lower rate of developing dementia than those randomized to placebo (HR, 0.47; 95% CI, 0.25–0.86) [27]. The results of the meta‐analysis of four studies showed that GLP‐1RAs users had a significantly lower risk of all‐cause dementia than non‐GLP‐1RAs users (RR, 0.72; 95% CI, 0.54–0.97); however, a high level of heterogeneity was found between studies (*I* ^2^ = 91.3%) [29].

This study has several limitations that warrant discussion. First, although exposure to GLP‐1RAs within the cohort was confirmed through claims data, the data lacked information on treatment adherence. This absence restricts our ability to fully evaluate the effect of treatment consistency on outcomes. Second, health services, preventive services, crucial laboratory data including blood sugar levels, haemoglobin A1c levels, renal and liver function markers, and biomarkers of dementia such as serum or cerebrospinal fluid neurofilament light chain, or scores of Clinical Dementia Rating‐Sum of Boxes, were not accessible within the secondary data utilised. However, because the data were population‐based, we assumed that there were no differences between the groups. Third, because this study relies on population‐based data from the Taiwan NHI programme and claims datasets, its generalisability to other countries may be limited. On the contrary, this study can be regarded as the first analysis focussing on Asian populations. Note that healthcare systems and patient populations vary across regions, potentially influencing treatment patterns and outcomes. Fourth, educational level appears to have been confirmed as potentially related to the incidence of dementia. However, due to limitations in the database, this study was unable to balance the differences between the experimental and control groups. Fifth, the mechanism by which GLP‐1RAs protect neurons in the brain remains unclear. In theory, the large‐molecule GLP‐1RAs currently available on the market cannot cross the blood‐brain barrier to directly provide neuroprotection. Recent studies suggest that GLP‐1RAs may indirectly protect neurons through improvements in glycaemic control, weight reduction, and physiological metabolism, thereby reducing the incidence of dementia. To mitigate these limitations, rigorous statistical adjustments were employed to account for potential differences between groups. However, given the inherent constraints of observational studies, further validation through randomized clinical trials is warranted to confirm the findings and establish causality.

Collectively, this study revealed that GLP‐1RAs may represent a promising new therapeutic approach to reducing the incidence of dementia, warranting further attention and in‐depth investigation of their cognitive‐protective potential in conditions such as T2DM and dementia. Careful evaluation of their risk–benefit ratio is necessary to establish an evidence‐based foundation for future clinical applications.

## Author Contributions

Hung‐Wen Cheng, Chiu‐Hsian Lee, and Gwo‐Ping Jong conceptualized the study and drafted the manuscript. Hung‐Wen Cheng and Gwo‐Ping Jong had significant input into data curation for the analysis. Hung‐Wen Cheng, Shun‐Fa Yang, Chiu‐Hsian Lee, and Gwo‐Ping Jong had significant contribution to the study methodology. Hung‐Wen Cheng, Shun‐Fa Yang, and Gwo‐Ping Jong had significant input into the investigation process. Hung‐Wen Cheng and Pei‐Lun Liao performed the formal analysis and visualized the results, and Gwo‐Ping Jong validated the analysis. All authors made significant contributions to the interpretation of the results and the review or editing of the manuscript. Hung‐Wen Cheng, Chiu‐Hsian Lee, and Gwo‐Ping Jong were responsible for project administration. Hung‐Wen Cheng acquired funding for this study. This study used data from the TBNHI Data Repository. Chiu‐Hsian Lee and Gwo‐Ping Jong are the guarantors of this work and, as such, had full access to all of the data in the study and takes responsibility for the integrity of the data and the accuracy of the data analysis.

## Ethics Statement

This study was approved by the ethics committee of Chung Shan Medical University Hospital (CS2‐24009). Written consent was not sought from the study participants as only de‐identified data were obtained from the TBNHI. The ethics committee waived the need for patient consent for this study.

## Conflicts of Interest

The authors declare no conflicts of interest.

## Peer Review

The peer review history for this article is available at https://www.webofscience.com/api/gateway/wos/peer-review/10.1002/dmrr.70058.

## Supporting information

Supporting Information S1

## Data Availability

The data used during this study are available from the corresponding authors on reasonable request.
