# Supplementary material for: Impact of Glucagon‐Like Peptide‐1 Receptor Agonists on the Dementia Incidence in Patients With Type 2 Diabetes Mellitus: A Population‐Based Longitudinal Cohort Study
Source: Diabetes Metab Res Rev. 2025 Jun 7;41(5):e70058. doi: 10.1002/dmrr.70058 (PMC12145076; doi:10.1002/dmrr.70058)
Supplement: Supplementary file 1 — Supporting Information S1 [file DMRR-41-e70058-s001.docx]

Supplemental

Table S1 ICD-9 / ICD-10 codes of all diagnosis.

| Diagnostic codes | ICD9-CODE/ ICD10-CODE |
| --- | --- |
| Hypertension | ICD-9 codes: 401.x-405.x,  ICD-10 codes: I10, I11.x, I13.x, I15.x, I16.x, I87.3x, I97.3x, O10.x, O11.x, O13.x, O16.x |
| Hyperlipidemia | ICD-9 codes: 272  ICD-10 codes: E78 |
| Heart failure | ICD-9 codes: 398.91, 402.01, 402.11, 402.91, 404.01, 404.03, 404.11, 404.13, 404.91, 404.93, 425.4–425.9, 428.x  ICD-10 codes: I50 |
| CKD | ICD-9 codes: 403.x, 404.x, 582.x, 583.0–583.7, 585.x, 586.x, 588.x  ICD-10 codes: E08.2x, E09.2x, E11.2x, E13.2x, I12.x, I13.1, N03.x, N04.x, N11.x, N18.x |
| Liver disease | ICD-9 codes: 070.22, 070.23, 070.32, 070.33, 070.44, 070.54, 070.6, 070.9, 456.0–456.2, 570.x, 571.x, 572.2–572.8, 573.3, 573.4, 573.8, 573.9, V42.7  ICD-10 codes: K72.x-K77, T86.43, T86.49, Z94.4 |
| COPD | ICD-9 codes: 490–492, 494-496  ICD-10 codes: J41-J44 |
| Malignancy | ICD-9 codes: 140-208  ICD-10 codes: C00-C96 |
| UTI | ICD-9 codes: 599.0, 595, 590  ICD-10 codes: N39.0, N30, N10, N11, N12, N13.6, N15.9, N16, N28.84 |
| Asthma | ICD-9 codes: 493  ICD-10 codes: J45 |
| CAD | ICD-9 codes: 410-414  ICD-10 codes: I20-I25 |
| Obstructive sleep apnea | ICD-9 codes: 327.20, 327.23, 327.29, 780.51, 780.53, 780.57  ICD-10 codes: G47.30, G47.33, G47.39 |
| Atrial fibrillation and flutter | ICD-9 codes: 427.3  ICD-10 codes: I48 |
| Alcohol-related disorders | ICD-9 codes: 291, 303, 305, 571.0, 571.1, 571.2, 571.3, 790.3  ICD-10 codes: F10, K70, R78 |
| Depression | ICD-9 codes: 296,300,309,311  ICD-10 codes: F30, F31, F32, F33, F34, F40, F41, F42, F43, F44, F45 |
| RA | ICD-9 codes: 7140  ICD-10 codes: M05, M06 |
| Dementia | ICD- 9-CM: 290.0, 290.1, 290.2, 290.3, 290.4, 294.1, 331.0,  ICD- 10-CM: F01, F02, F03, G30, G31 |
| Stroke | ICD-9 codes: 430-435  ICD-10 codes: I60, I61, I62, I63, I65, I66, I67.84, G45, G46, |

CKD: chronic kidney disease; chronic obstructive pulmonary disease; UTI: urinary tract infection; CAD: coronary artery disease; RA: rheumatoid arthritis

Table S2 Anatomical Therapeutic Chemical (ATC) codes of all drugs

| Medications | ATC code |
| --- | --- |
| NSAIDs | M01A, M02AA |
| Corticosteroids | H02 |
| Aspirin | N01AC06, N02BA01, B01AC06 |
| Statin | C10AA, C10B |
| Biguanides | A10BA |
| Sulfonylureas | A10BB |
| Alpha glucosidase inhibitors | A10BF |
| Thiazolidinediones | A10BG |
| DPP-4 inhibitors | A10BH |
| Insulin | A10A |
| SGLT-2 inhibitors | A10BK |
| Alpha-blockers | C02CA |
| Beta- blockers | C07 |
| CCB | C08 |
| ACEI | C09A, C09B |
| ARB | C09C, C09D |

NSAIDs: Nonsteroidal anti-inflammatory drugs; DPP-4 inhibitors: Dipeptidyl peptidase-4 inhibitors; SGLT-2 inhibitors: Sodium-glucose co-transporter 2 inhibitors; CCB: Calcium channel blockers; ACEI: Angiotensin-Converting Enzyme Inhibitors; ARB: Angiotensin II receptor blockers
